# Supplementary material for: Considering patient clinical history impacts performance of machine learning models in predicting course of multiple sclerosis
Source: PLoS One. 2020 Mar 20;15(3):e0230219. doi: 10.1371/journal.pone.0230219 (PMC7083323; doi:10.1371/journal.pone.0230219)
Supplement: S1 Table — (PDF) [file pone.0230219.s001.pdf]

Supplementary Table S1. Confusion Matrices for the Visit-Oriented setting

	**Feature-saving**		**Record-saving**	
**180 days**				
SVM	3709	585	12834	1882
	2	34	31	176
RF	3708	586	12513	2203
	0	36	19	188
AB	3691	603	12558	2158
	0	36	23	184
KNN	3116	1178	12612	2104
	7	29	39	168
**360 days**				
SVM	3544	621	12160	1871
	5	32	40	167
RF	3636	529	11662	2370
	4	33	24	183
AB	3561	604	11719	2312
	6	31	24	183
KNN	2967	1198	11936	2095
	12	25	47	160
**720 days**				
SVM	3297	589	11404	1567
	7	30	47	160
RF	3353	533	11183	1788
	8	29	33	174
AB	3384	502	11032	1939
	11	26	32	175
KNN	2922	964	11072	1899
	13	24	50	157

SVM: Support vector machines; RF: Random Forest; AB: Ada Boost; KNN: K nearest neighbours
