# Supplementary material for: Considering patient clinical history impacts performance of machine learning models in predicting course of multiple sclerosis
Source: PLoS One. 2020 Mar 20;15(3):e0230219. doi: 10.1371/journal.pone.0230219 (PMC7083323; doi:10.1371/journal.pone.0230219)
Supplement: S2 Table — (PDF) [file pone.0230219.s002.pdf]

**Supplementary Table S2**. Confusion Matrices for the History-Oriented setting

| **Feature-saving** | | **Record-saving** | |
|---|---|---|---|
| **180 days** | | | |
| 954 | 34 | 3736 | 45 |
| 5 | 4 | 32 | 20 |
| **360 days** | | | |
| 941 | 23 | 3469 | 62 |
| 6 | 4 | 26 | 26 |
| **720 days** | | | |
| 890 | 23 | 3160 | 47 |
| 4 | 6 | 17 | 35 |

All data were obtained using LSTM model
