# Supplementary material for: Considering patient clinical history impacts performance of machine learning models in predicting course of multiple sclerosis
Source: PLoS One. 2020 Mar 20;15(3):e0230219. doi: 10.1371/journal.pone.0230219 (PMC7083323; doi:10.1371/journal.pone.0230219)
Supplement: Attachment — Submitted filename: Rebuttal.pdf [file pone.0230219.s003.pdf]

Detailed responses to Journal requirements and Reviewer's comments.
Line numbers are relative to the annotated version

RESPONSES TO JOURNAL REQUIREMENTS:

1. Please ensure that your manuscript meets PLOS ONE's style requirements, including those for file naming.
The manuscript has been prepared using the provided templates.

2. In the Methods section and the online submission form, please provide additional information about the patient records used in your retrospective study. Specifically, please ensure that you have discussed whether all data were fully anonymized before you accessed them and/or whether the IRB or ethics committee waived the requirement for informed consent. If patients provided informed written consent to have data from their medical records used in research, please include this information.
Anonymization is described at lines 137-138; the written consent is mentioned at lines 115-116.

3. We note that you have stated that you will provide repository information for your data at acceptance. Should your manuscript be accepted for publication, we will hold it until you provide the relevant accession numbers or DOIs necessary to access your data. If you wish to make changes to your Data Availability statement, please describe these changes in your cover letter and we will update your Data Availability statement to reflect the information you provide.
We have uploaded the datasets used to FigShare, link will be provided upon acceptance of the paper. Please note that we have removed the labels to most features, to protect privacy of study participants to the maximum possible extent. This action will not preclude duplication of our results.

4. Please ensure that you have provided details of the software used to implement the machine learning algorithms used in your study.
The software is indicated at lines 231 and 273-274.

RESPONSES TO REVIEWER 1
1. I would like the authors to be more explicit on the specific factors that predicted SPMS, and how that compares to the literature on predictors of SPMS. The authors should provide a brief review of that literature, to put this article into context.
Thank you for your suggestion. Several sentences and references have been added to comply with your request. In particular, we have modified the manuscript at lines: 7-31; 41-42; 188-191. The references quoted in these paragraphs have been added.

2. Was there any subgroup analysis performed?
Unfortunately, our sample was too small for reliable subgroup analysis.

3. Are there plans for an independent validation set?
Thank you for raising this important point. We are currently working to extend our analysis to larger cohorts. The possible future development of the present work are now indicated at lines 104-108.

4. Two years is not much of a lag phase; is there any suggestion from records review that these patients had entered the SP stage before it was officially recognized?

Of course, SP phase is diagnosed retrospectively, and some delay is possible. However, we believe that the database is reasonably accurate, as patients' records are updated at every visit, and the disease phase can be revised if the attending neurologist thinks it is adequate to do so. This is now indicated at lines 120-121.

5. What is the difference between 2nd line and immunosuppressant therapy? Please define these treatments (including 1st line).
The treatments are now defined at lines 161-170.

6. What is the transition rate to SPMS so low (13% over 40 years) in this cohort?
Thank you for your observation. This important point is discussed at lines 124-133.
